# Commentary: Telephone-based nursing as a means to improve health and self-efficacy in chronic obstructive pulmonary disease

**DOI:** 10.1177/17449871251370624

**Published:** 2025-09-17

**Authors:** Pernilla Garmy

**Affiliations:** Professor, Kristianstad University, Sweden

Commentary on: Effect of telenursing empowerment programme on self-efficacy and health status of patients with COPD(Rajabi et al., 2025).

Living with chronic obstructive pulmonary disease (COPD) entails considerable suffering for the individual and poses a significant burden on both society and the healthcare system. Many people with COPD experience physical limitations, recurrent exacerbations, anxiety; and depression; all of which negatively affect their quality of life. To address these complex needs, healthcare must be flexible and person-centred – particularly when supporting individuals with chronic conditions.

In the present study (Rajabi et al., 2025), the authors investigated the effects of a telenursing-based empowerment programme, where nurses provided support to patients with COPD through 14 scheduled telephone calls over a period of 7 weeks following hospital discharge. The educational intervention covered a wide range of topics related to self-care and disease management, including understanding lung function and disease pathology, available treatments, medication use, nutrition, balancing activity and rest, physical training, smoking cessation, use of inhalers and oxygen equipment, prevention of pneumonia, and physiotherapy exercises to perform at home.

The study was designed as a randomised controlled trial including 108 patients who were randomly assigned to either an intervention or control group. Participants’ levels of self-efficacy and health status were measured at baseline, immediately after the intervention, and again at 3-month follow-up. The results demonstrated that the intervention group had significantly improved levels of self-efficacy and perceived health at follow-up.

These findings are important, as they suggest that a structured and individualised nurse-led telephone intervention can effectively support patients in their self-care and have long-term positive effects. This is particularly valuable in today’s context of limited access to healthcare professionals and increasing reliance on home-based care.

Similar conclusions have been drawn in a recent systematic review and meta-analysis by Mun et al. (2024), which examined the effects of telenursing in home healthcare for chronic conditions such as COPD and heart failure. That review found that telenursing was associated with fewer hospital admissions, fewer emergency department visits, and improved quality of life. The authors highlighted telenursing as a promising approach for supporting self-care and reducing healthcare utilisation – particularly in community-based care.

In summary, this study demonstrates that nurse-led telephone-based interventions, when delivered in a systematic and patient-centred way, can strengthen patients’ ability to manage their disease and improve both perceived health and overall well-being. It provides a valuable contribution to the growing field of eHealth and person-centred care for people living with COPD.

## References

[bibr1-17449871251370624] Mun M Park Y Hwang J , et al. (2024) Types and effects of telenursing in home health care: A systematic review and meta-analysis. Telemedicine Journal and e-Health 30: 2431–2444. DOI: 10.1089/tmj.2023.0188. 37707998

[bibr2-17449871251370624] Rajabi S Momeni M Ranjbaran M , et al. (2025) Effect of telenursing empowerment programme on self-efficacy and health status of patients with chronic obstructive pulmonary disease: randomised controlled trial. Journal of Research in Nursing 30: 421–436. DOI: 10.1177/17449871241308826 PMC12170556 40535615

